# Biological Properties of Calcium Phosphate Bioactive Glass Composite Bone Substitutes: Current Experimental Evidence

**DOI:** 10.3390/ijms20020305

**Published:** 2019-01-14

**Authors:** Maria Karadjian, Christopher Essers, Stefanos Tsitlakidis, Bruno Reible, Arash Moghaddam, Aldo R. Boccaccini, Fabian Westhauser

**Affiliations:** 1 Center of Orthopedics, Traumatology, and Spinal Cord Injury, Heidelberg University Hospital, Schlierbacher Landstr. 200a, 69118 Heidelberg, Germany; maria.karadjian@med.uni-heidelberg.de (M.K.); christopher.essers@med.uni-heidelberg.de (C.E.); stefanos.tsitlakidis@med.uni-heidelberg.de (S.T.); bruno.reible@gmail.com (B.R.); 2 ATORG—Aschaffenburg Trauma and Orthopedics Research Group, Center for Trauma Surgery, Orthopedics, and Sports Medicine, Klinikum Aschaffenburg-Alzenau, Am Hasenkopf 1, 63739 Aschaffenburg, Germany; Arash.Moghaddam-Alvandi@klinikum-ab-alz.de; 3 Institute of Biomaterials, University of Erlangen-Nuremberg, Cauerstr. 6, 91058 Erlangen, Germany; aldo.boccaccini@ww.uni-erlangen.de

**Keywords:** calcium phosphate, bioactive glass, bone substitutes, composite bone substitute materials, bone tissue engineering

## Abstract

Standard treatment for bone defects is the biological reconstruction using autologous bone—a therapeutical approach that suffers from limitations such as the restricted amount of bone available for harvesting and the necessity for an additional intervention that is potentially followed by donor-site complications. Therefore, synthetic bone substitutes have been developed in order to reduce or even replace the usage of autologous bone as grafting material. This structured review focuses on the question whether calcium phosphates (CaPs) and bioactive glasses (BGs), both established bone substitute materials, show improved properties when combined in CaP/BG composites. It therefore summarizes the most recent experimental data in order to provide a better understanding of the biological properties in general and the osteogenic properties in particular of CaP/BG composite bone substitute materials. As a result, BGs seem to be beneficial for the osteogenic differentiation of precursor cell populations in-vitro when added to CaPs. Furthermore, the presence of BG supports integration of CaP/BG composites into bone in-vivo and enhances bone formation under certain circumstances.

## 1. Introduction

Bone defect augmentation belongs to the clinically most important procedures, not only in orthopedic surgery, but also in the overall context of modern medicine: With two million procedures annually, bone grafting is the second most performed tissue transplantation in the United States after blood transfusion [1]. The current gold standard of bone defect repair remains autologous bone grafting, mostly harvested from the iliac crests [2]. This biological reconstruction of bone is described as bone tissue engineering [3]. However, defect treatment and bone tissue engineering using autologous tissue is not only restricted by the available bone substance, it also requires a second intervention that might be followed by surgical site complications [4,5]. Therefore, the development, evaluation and production of synthetic bone substitutes that can either limit or even replace the usage of autologous bone marrow as a grafting material is in the spotlight of experimental and clinical orthopedic research. The aim is to create synthetic bone substitutes exhibiting an intrinsic osteogenic activity and morphological features that are comparable to iliac crest bone as grafting material [6,7,8].

The mentioned requirements for synthetic bone substitute materials can be summarized as their “biological properties”—a term that has to be defined prior to use within this review paper. From a bone tissue engineering perspective, the term “biological properties” summarizes the influence of the respective material towards cell viability, cell proliferation, and immunogenic reaction, i.e., the biocompatibility and bioactivity [9]. However, not only biocompatibility is a requirement for bone substitutes. Specifically, their influence on osteogenic (which can be described as osteostimulation) and angiogenic differentiation, as well as osseointegration and osteoconduction are of certain importance [3,8].

In experimental settings, the biological and/or osteogenic properties of bone substitute materials are evaluated using certain in-vitro culture settings and in-vivo models. The in-vitro models mostly focus on the evaluation of cell-material contact (adherence), biocompatibility of the materials, the influence of the material itself or of soluble parts of the material on cell vitality, proliferation, and/or differentiation [10,11,12,13]. In-vivo models can either be used as bioreactors when the bone substitutes are implanted ectopically in the host organism, providing nutrition of the implant, or as actual orthotopic bone defect models [7,14]. Ectopic models mostly provide analysis of biocompatibility, vascularization and osteoid formation, orthotopic models also allow for analysis of (amongst others) mechanical properties, osseointegration and osteoconduction [7,14,15].

The most commonly used synthetic bone substitutes to date are calcium phosphates (CaPs), mostly as derivatives of hydroxyapatite (HA; Ca_10_(PO_4_)_6_(OH)_2_) and tricalcium phosphate (TCP; Ca_3_(PO_4_)_2_) [8,16,17]. Whilst the osteoconductive properties of CaPs are good, the material itself shows limited stimulation of osteogenic differentiation and surface reactivity is comparably low [16,18,19]. In clinical routine, CaPs suffer from the problem of either too fast or too slow resorption, again impairing biological properties: Slow resorption inhibits osseointegration, whereas fast resorption might lead to insufficient “filling” of the treated bone defect [8,20].

An attractive alternative to CaPs as bone substitute materials are bioactive glasses (BGs): BGs are osteostimulative and they exhibit formation of a carbonate-substituted hydroxyapatite-like (HCA) layer on their surfaces both in-vitro and in-vivo, providing bonding to bone and surrounding tissues [9,21]. Furthermore, BGs are proven to stimulate angiogenic and osteogenic differentiation of stem cells by release of bioactive ions [22,23,24]. It is therefore possible to tailor the properties of BGs towards specific needs: For example, boron can be added to the BG composition to improve angiogenic properties [22]. The most commonly used BG is the 45S5 “Bioglass” with a composition of 45% SiO_2_, 24.5% Na_2_O, 24.5% CaO, and 6% P_2_O_5_ (in wt%) [25]. 45S5-BG provides strong bonding to surrounding tissues and has shown osteogenic capabilities, making it a class-A-biomaterial [25,26]. However, 45S5-derived BGs suffer from poor mechanical properties when used as three-dimensional (3D) bone substitutes: The 45S5-BG has the tendency to crystallize during heating procedures when producing 3D scaffolds. As a consequence, stability decreases, making 3D scaffolds brittle [27,28,29,30,31]. Another limitation of the 45S5-BG, especially when used in in-vitro experimental settings, is caused by the high Na_2_O-portion within the glass composition. In contact with (body) fluids, Na_2_O dissolves, causing a liberation of sodium ions followed by a strong pH-increase that might be harmful to cells [32,33]: Whilst osteoblast function is stimulated by a slightly alkaline milieu, the osteoclast function is decreased by alkaline surroundings [34,35,36]. Since osteoclasts are of certain importance in the early phases of bone regeneration, the pH-increase induced by 45S5-BG might limit the initial steps of osseous regeneration, especially when using “pure” 45S5-BG scaffolds [25,37,38]. Therefore, prior to the use of 45S5-derived BG scaffolds in in-vitro studies, pretreatment periods are necessary to diminish the initial burst release of ions resulting in a dramatic increase of pH [39,40,41].

There are approaches to improve the osteogenic properties and to overcome the limitations of both material types by combining CaPs and BGs creating CaP/BG composite materials. Bellucci et al. extensively reviewed the current literature concerning CaP/BG composites with a focus on material development, characteristics, and properties [16]. However, a systematic review of literature focusing on biomedical and osteogenic features of CaP/BG composite bone substitutes is not yet available.

This structured review summarizes the most recent relevant experimental methods and data in order to provide a better understanding of the biological properties in general and the osteogenic properties of CaP/BG composite bone substitute materials.

## 2. Methods

A structured review in the sense of a meta-analysis was conducted according to the PRISMA-guidelines by screening the PubMed database in December 2018 [42]. The MeSH Term “Calcium Phosphates” and the term “Bioglass” were applied leading to 214 results. The MeSH Term “Calcium Phosphate” summarizes the keywords “apatites”, “hydroxyapatites”, and “calcium phosphates”, the term “Bioglass” summarizes the different bioactive glass compositions. The titles and abstracts of all results were screened according to the defined inclusion criteria: original works and biological evaluation of CaP/BG composites compared to each other or to CaP or BG respectively, designed for orthopedic application regarding osteogenic properties in-vitro and in-vivo. Exclusion criteria were: Failed matching with the inclusion criteria, (metal) implant coatings consisting of CaP/BG composites, missing full-texts and full texts not available in English language. 200 publications did not match the inclusion criteria and therefore were excluded. 14 publications were included and went into meta-analysis. Since the experimental approaches found in the studies differed significantly, meta-analysis (i.e., comparative quantification) was carried out as precisely as possible. However, direct quantification and comparison of the data found was not accurately possible.

## 3. Overview

Table 1 gives an overview of the included studies summarizing the material composition, the methods used for biological characterization of the respective materials as well as the biological outcome.

## 4. The Rationale behind CaP/BG Composite Materials for Application in Bone Tissue Engineering

Bellucci et al. published a comprehensive review about CaP/BG composites in 2016, with a main focus on the material properties [16]. They identified two main motivations for the production and application of CaP/BG composites: firstly, the possibility to tune the dissolution and the resorption behavior of the CaP in order to achieve superior biological properties [1,16,19]. The ratio of resorption and tissue remodeling and therefore the formation of bone tissue within the biomaterial, its integration into the bone, and the replacement of the material by vital bone tissue is one of the key properties making a bone substitute “attractive” [1]. Mainly for TCP-based scaffolds, strong resorption might end in overly fast chemical and cellular degradation, leading to insufficient ingrowth of bone tissue resulting in empty defect sites that remain unconsolidated [1,54]. Secondly, by addition of BGs to CaPs as a sintering aid, the resulting composite material exhibits improved mechanical properties compared to either BGs or CaPs alone [19].

From a tissue engineering perspective, by addition of BGs to CaP-based bone substitutes the positive features of both materials might be combined, whilst the negative features could be limited in the same way [29]. For example, the initial pH-increase of “pure” 45S5-derived scaffolds can be limited by adding a certain amount of BG to CaPs, creating a CaP/BG composite scaffold material [9,16,20,24].

Another attractive feature of BGs is the surface reactivity, which defines the bioactivity of the material [9,26,33,55]. Compared to CaPs, the bioactivity of BGs is higher [16,56]. A combination of both materials might thus also improve the biological properties, including stimulation of precursor cells towards osteogenic differentiation, enhanced angiogenesis and a positive influence on cell viability and proliferation [16,55].

## 5. CaP-Types Used as Part of the Composite and Their Influence on Biological Properties

Within the collective of studies included into the meta-analysis, in six cases HA was used [18,19,47,48,49,52]. Also in six studies, β-tricalcium phosphate (β-TCP) was used as the CaP-part of the composite material [43,45,47,50,51]. In three cases, a mix of HA and β-TCP was used: Lu et al. described that the HA/β-TCP mix in a ratio of 40:60 serving as CaP part of the scaffolds comes closer to the physiological composition of bone and therefore exhibits improved biocompatibility [46]. In the study of Bernhardt et al., several CaP compositions were used, including pure HA and β-TCP as well as a HA/β-TCP mix with a ratio of 60:40 [47]. Interestingly, the study focused on evaluation of the osteogenic properties of materials that are already available for clinical use. Barbieri et al. used a combination of β-TCP and HA in a ratio of 96:4. However, the HA/β-TCP mix was not combined with BG but with an alkylene oxide copolymer (AOC) [43].

HA, a highly crystalline form of CaP, shows excellent biocompatibility, coming very close to the inorganic extracellular mineralized phase of bone [8,16,17]. Mostly used as a porous scaffold material, HA exhibits osteoconductive features with limited osteostimulation together with a slower rate of resorption compared to other CaPs such as TCP [20]. HA tends to be brittle, especially with increasing porosity of scaffolds [20]. The most commonly used TCP is β-TCP, showing faster resorption rates compared to HA and exhibiting better mechanical features [20]. However, the faster rate of resorption of TCP also limits the mechanical properties when implanted into bone defects: Due to the fast degradation, defects might not be filled by newly formed bone tissue and therefore remain empty causing mechanical deficits [8,20]. Furthermore, the bonding of surrounding tissues to CaPs is not very strong, thus the surfaces only insufficiently provide HCA formation [56]. HCA formation is directly linked to strong bonding of bone substitutes to the surrounding tissues [9,21,33]. In the study of Chen et al. Si-Sr-Zn-Mg-codoped CaP was used, but the exact type of CaP was not specified [44]. Haimi et al. also did not specify the used CaP [53].

## 6. BG Compositions Used

Hench and coworkers developed the first BG in the late 1960s [57]. Since then, the family of BGs grew rapidly [24,26,57]. BGs have been designed to provide certain biological properties: Added as a second phase to CaPs these BGs can provide features that tailor CaPs towards a specific (clinical or experimental) field of use [10,26,57]. A combination of CaPs and BGs in the sense of CaP/BG composite materials might therefore be a way to combine positive aspects of both materials and—from a biological perspective—to improve cell-material interaction as well as the differentiation of precursor cells towards osteogenic lineage.

45S5-BG was the most used BG type within the included studies: Five studies used 45S5-BG as the BG part of the composite material (Table 1) [43,44,45,48,52]. Three studies used a glass based on the BG_Ca (47.3% SiO_2_, 45.6% CaO, 4.6% Na_2_O, 2.6% P_2_O_5_) [18,19,48]. This BG exhibits improved mechanical properties compared to 45S5-BG due to lower sintering temperatures limiting crystallization of the glassy phase during processing [16,19,48].

Some studies used BGs based on phosphorous pentoxide without silica content [51]: Yu et al. described that silicon-free glasses come closer to the composition of natural bone and also provide good bioactive properties [51]. The group of Cholewa-Kowalska used BGs with either high (S2) or low (A2) silica content [49]. Their aim was to tailor the bioactivity of the glasses within the resulting composites, as lower silica content positively correlates with bioactivity [26,49].

## 7. In-Vitro Evaluation Models

**Figure 1:**
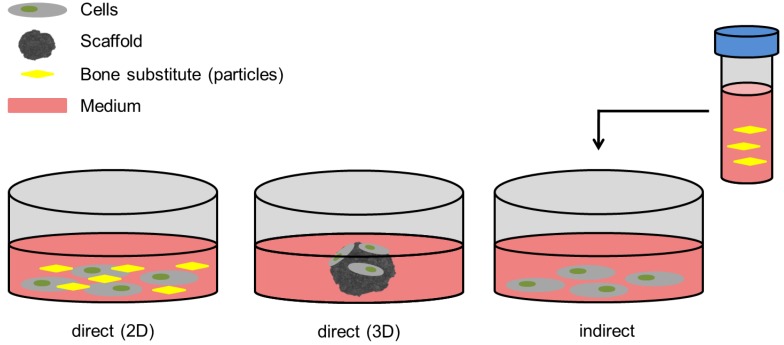
In-vitro evaluation models. 2D culture allows physical contact, mostly with particles of the respective bone substitute, whilst cells seeded on scaffolds are cultivated in 3D culture conditions. The indirect culture setting allows evaluation of the dissolution products of the materials that underwent incubation in the respective medium for a certain amount of time before the particle-free medium is transferred to the cells (indicated by the black arrow).

Out of the total number of 14 studies that were included in the review, in-vitro approaches were used in 10 studies (71%). Eight of 10 studies in the in-vitro setting were performed in 3D or two-dimensional (2D) “direct” culture settings [45,46,47,48,49,50,51,53]. The cells were either seeded directly onto scaffolds (3D culture) or incubated in physical presence of and in contact to parts of the materials (2D culture setting, Figure 1). Within the remaining studies, additional “indirect” incubation was used [19] (Figure 1), while Chen et al. were the only group using the indirect setting exclusively [44]. This approach is specified by incubation of the material and only the material in the cell culture medium for a certain amount of time, allowing the ionic or chemical components of the material to dissolve into the medium, creating a “saturated medium” [10]. The cells are then exposed to the saturated medium without being physically exposed to the actual material. Both approaches are common ways to evaluate the very basic properties of bone substitute materials, though it has been shown that the dissolution products and the direct contact of cells to the material induce changes in cell metabolism and/or proliferation [10,58].

Whilst the indirect approach allows a detailed evaluation of the influence of the material’s composition on the cells, direct culture settings also take the interaction of cells and material morphology into account [10,58]. Possible limitations for the use of direct culture settings are the high bioactivity of BGs that might lead to cell death and therefore requires careful pre-treatment of the used materials prior to contact with cells [41]. On the other hand, 3D culture settings in-vitro are challenging: Nutrition of cells, even in well-oxygenated surroundings, is only warranted by diffusion, which is a comparatively slow and insufficient type of nutrition compared to either dynamic, perfusion-based approaches or in-vivo settings with a focus on vascularization [13,14,59]. It was demonstrated that static 3D in-vitro culture settings are not sufficient to support stable cell populations, resulting in decreasing cell numbers—a limitation that was not yet described for indirect approaches [13,58]. Indirect cultivation of cells in presence of soluble parts of the materials under static conditions might therefore also help to assess the long-lasting differentiation characteristics of cells: Whilst the impact of the materials on cell proliferation and/or viability can be analyzed within a few days, osteogenic differentiation of cell populations takes weeks [60,61,62].

An example for “indirect” testing is the study of Chen et al.: Tests were performed with cells that were in indirect contact with the bone grafting material through “conditionized cell culture medium”, which was produced by immersion of the grafting material in Dulbecco’s modified eagle medium (DMEM) for 48 h [44]. Bellucci et al. used a combination of direct and indirect evaluation methods: Cell viability and proliferation were assessed indirectly, while the cells were also directly exposed to the material for the evaluation of the material’s cytotoxicity; cell morphology was evaluated both after direct and indirect contact [19]. Although the authors described that there were no morphological differences between cells cultured in direct or indirect contact to the composite material, there is some evidence that the physical presence of materials (in this case BGs) has an impact on cell viability and proliferation, with a positive correlation of concentration and decreased cell vitality [58]. Therefore, the culture conditions have to be set carefully in order to meet the respective requirements and to specifically focus on the hypothesis of a study.

The cells used for the in-vitro assessment of bone grafting materials vary widely between the studies: Not only different cell types and cell numbers were used, but also cells originating from different species. In Table 2, the cell types, species, numbers, and culture settings are therefore summarized.

According to ISO-standards for biological evaluation of medical devices 10993-5, most of the studies (six of 10) used established cell lines to perform their assays [19,45,47,48,50,51]. Four studies used cells of primary cell cultures, which is feasible according to the ISO-standards if “[…] reproducibility and accuracy of the response can be demonstrated” [44,46,49,53]. However, it was shown that the in-vitro studies used not only different cell culture settings but also different cell types of different species at different cell numbers that they incubated in different cell culture media, which means that practically every culture condition parameter differed between the studies. This complicates the interpretation of the studies and significantly reduces direct comparability.

### 7.1. Analysis of Cell Morphology, Adhesion and Surface Interaction

Cell morphology, adhesion on the scaffold and cell-surface-interaction were evaluated by scanning electron microscopy (SEM) in six studies [45,46,47,48,51,53], while Bellucci et al. evaluated cell morphology of cells having direct or indirect contact to bone grafting materials with conventional optical microscopy [19]. The assessments were performed at different time points serving different purposes: Cell attachment/adhesion was evaluated rather early in cell culture [19,45,46,47,53] whereas cell growth, material–cell interaction and cell morphology was evaluated after 14 [51,53], 21 [48] or 28 days [47] of cell culture. Viable cells were detectable after seeding on scaffolds/incubating in medium that was in direct contact with the material in all studies which is an important prerequisite before performing further evaluations corresponding to the ISO-standards. Microscopy is a method for assessing cell well-being while offering an individual evaluation of materials and their particular interactions with cells. A disadvantage of microscopy is the difficulty of quantification of the results, rather allowing qualitative or semi-quantitative analyses. Still, evaluation of cell well-being during culture is crucial for performing further assays, especially cytotoxicity assays (ISO 10993-5).

### 7.2. Analysis of Cell Viability, Cytotoxicity, and Proliferation

The majority of the in-vitro studies (72%) evaluated cell viability and metabolic activity quantitatively using colorimetric enzyme-dependent assays (e.g., the 3-(4,5-dimethylthiazol-2-yl)-2,5-diphenyltetrazolium bromide (MTT) test) [19,44,45,46,47,48,49,50]. Every test consists of a substrate that is transformed into a colorimetrically detectable product by intracellular (mitochondrial) enzymes—the turnover (measured spectrophotometrically) correlates with the metabolic activity of the cells and with the number of viable cells [63]. Cell proliferation cannot be specifically derived from the colorimetric assays, so several additional methods were used to evaluate the proliferation: Bellucci et al. performed a bromodeoxyuridine-test [19], while Bernhardt et al. performed a dsDNA (double stranded deoxyribonucleic acid) quantification assay as well as the determination of lactate dehydrogenase (LDH) activity as proliferation marker for viable cells [47]. Others observed cell proliferation qualitatively by performing SEM of cell seeded scaffolds at two time points [46,47]. Cai et al. were the only group who evaluated only the proliferative capacity of the cells without monitoring their viability: They assessed cell proliferation by dislodging adherent cells from scaffolds and manually counting them in a blood cell counting chamber at three different time points [51]. Haimi et al. quantified the DNA content and performed a live/dead fluorescent cell staining at two different time points to determine cell proliferation both quantitatively and qualitatively as well as cell viability qualitatively through fluorescence microscopy [53]. The colorimetric assays allow to assess cytotoxicity of the material, as cytotoxic materials reduce cell number by causing cell death and diminishing metabolic activity of the surviving cells. Nevertheless, Bellucci et al. included an additional assay (Neutral Red Uptake Test (NRU)) to evaluate cell viability as a correlate to cytotoxicity of the material in direct contact to the cells [19]. Except for the studies of Cai et al. and Haimi et al. [51,53], every group performed an ISO-conform cytotoxicity test (meaning NRU, MTT, XTT (2,3-bis(2-methoxy-4-nitro-5-sulfophenyl)-5-[(phenylamino)carbonyl]-2H-tetrazolium hydroxide)), or a comparable test [48] to evaluate cytotoxic potential of the material. A particular protocol was used by Bellucci et al., who pre-incubated the cell-seeded scaffolds in growth medium for 15 days prior to incubating them in osteogenic differentiation medium [48]. Cell proliferation tests using the AlamarBlue Assay were performed after two, seven, and 14 days of incubation in osteogenic differentiation medium, resulting in a longer total incubation time of the cells on the scaffolds [48].

### 7.3. Analysis of Osteogenic Differentiation

Six of the 10 in-vitro studies assessed osteogenic differentiation, all by means of different methods, for example by measuring the activity of alkaline phosphatase (ALP) as a marker enzyme of osteoblasts correlating with the osteogenic differentiation of a cell population [44,46,47,48,49,53]. The evaluation of the activity of ALP is a valid method to measure osteogenic differentiation, as the enzyme is very active in osteoblastic cells; precursors of osteoblasts (preosteoblasts) can even be recognized by membrane-associated alkaline phosphatase markers in-vivo [64,65,66,67]. However, since the osseous alkaline phosphatase activity is rather unstable regarding temperature changes, assays need to be processed in highly standardized protocols to guarantee comparability of the results [68]. Nevertheless, determining ALP activity remains a gold standard parameter for osteogenic differentiation. Haimi et al. semi-quantitatively determined ALP activity by additionally performing a leukocyte ALP staining of the cell-seeded scaffolds, which visualizes that the photometrically detected activity originates from the ASC, as ALP is expressed in other cells as well [53,69].

In some studies, evaluation of ALP activity is combined with quantification of gene expression using quantitative real-time polymerase chain reaction analysis (qPCR) [44,46,47]. By quantifying the expression of osteogenic genes, osteogenic differentiation can be monitored in greater detail: Different genes are highly expressed at different maturation stages of osteoblastic cells [70]. A detailed observation of the cellular differentiation is therefore possible by using different combinations of early and late osteogenic genes [71]. The measurement of gene activity can be complemented by quantifying the expression of proteins that are encoded by the respective genes. For example, Chen et al. used an Enzyme-linked Immunosorbent Assay (ELISA) to quantify protein production [44]. Haimi et al. were the only group to perform an assay to measure the concentration of osteopontin, a specific protein crucial for bony development, without evaluating the corresponding gene expression [53].

As another correlate of osteogenic differentiation, cell populations that develop towards osteoblasts start to build a certain extracellular matrix that includes calcium deposits and therefore undergoes mineralization [44,60,61]. Alizarin Red S-staining is a method to evaluate mineralization in-vitro, however it needs to be interpreted carefully when used in 2D and 3D direct culture (thus in physical presence of CaPs and BGs): Especially when analyzing BGs that start building a HCA-layer in physiological solutions, the resulting calcium deposition might lead to false positive results even without osteogenic differentiation and matrix deposition of the surrounding cell population [25,33,60,61,72]. In the present study of Chen et al., Alizarin Red S-stained samples did not contain scaffolds, but cells in culture medium that was in direct contact with the material [44]. Therefore, the staining can be used as a valid method for evaluating mineralization [44,73,74,75].

### 7.4. Cell Culture Media Used

Although the cell culture media also differ between the studies, it is possible to classify two groups in dependency on the study objective: In six studies, maintenance medium without any factors stimulating osteogenic differentiation was used, focusing on cell proliferation and viability and trying to prove the absence of cell cytotoxicity induced by the respective material [19,45,46,50,53,76], including rather short cultivation periods (median seven days, range from 24 h to 28 days). In four studies the cells were incubated in medium including factors promoting osteogenic differentiation [44,47,48,49]. These studies focused not only on cell proliferation and cytotoxicity of the material, but also on the osteoinductive potential of the material, meaning the osteogenic differentiation of the cells.

In two studies, β-glycerophosphate and ascorbic acid were added to the maintenance medium [44,47], while Cholewa-Kowalska et al. added ascorbic acid and dexamethasone [49]. It is known that dexamethasone, ascorbic acid, and β-glycerophosphate stimulate osteogenic differentiation in in-vitro settings by influencing different cellular pathways required for differentiation of mesenchymal stem cells (MSCs) to osteoblasts [60,61,77]. It is shown for dexamethasone that at least three weeks of continuous stimulation are necessary to reach full osteogenic differentiation, while only Bernhardt et al., Bellucci et al. and Chen et al. incubated for at least three weeks [44,47,48,78]. In the study of Cholewa-Kowalska et al., osteogenic differentiation medium was used, while the only assessed osteogenic differentiation marker was an ALP activity assay after seven days of cell culture [49]. Keeping the required stimulation time and continuity of osteogenic stimulation by dexamethasone in mind, it does not seem reasonable to evaluate osteogenic differentiation at a single time point after a short stimulation [79]. Cholewa-Kowalska additionally compared the cell number of samples incubated in maintenance medium (MM) to samples incubated in osteogenic differentiation medium (ODM) [49]. Bellucci et al. pre-incubated MC3T3-E1 cells in growth medium in order to allow proliferation before changing the medium by adding the osteostimulating factors β-glycerophosphate and ascorbic acid [48].

The other studies were using media without stimulating factors, aiming to show the osteostimulative capabilities of the material itself, as osteogenic stimulation factors are not necessary to evaluate cell survival and proliferation. The necessity and justification of the usage of osteogenic differentiation factors therefore depends on the assays that shall be performed—while their usage is justified in studies evaluating the osteogenic differentiation (e.g., by gene expression analysis), they are not required in studies assessing material properties such as cytotoxicity.

Lu et al. additionally compared the effect of pre-stimulation of the ASCs with BMP-2 on osteogenic differentiation to non-stimulated ASC, both cultured in growth medium [46]. They found that in the pre-stimulated group osteogenic differentiation (evaluated by means of qPCR and ALP activity) was significantly higher than in the unstimulated group, thus cells with two differentiation statuses were included in the study.

## 8. In-Vivo Evaluation Models

Out of the total number of 14 studies that were included into the review, in-vivo approaches were used in four studies (27%). The respective in-vivo protocols are summarized in Table 3. No study used both in-vitro and in-vivo protocols. Three of these four in-vivo studies for the evaluation of bone graft composites used a New Zealand Rabbit animal model [18,51,52]. The group of Barbieri et al. used dogs as host organisms [43]. Every in-vivo study used an orthotopic design, thus the bone substitute material was implanted in bone defects. In the study of Barbieri et al., an ectopic intra-muscular implantation model was used additionally [43].

The implantation time varied among the studies in a range between one and six months, while two studies included multiple time points [51,52]. Each of the presented in-vivo settings included histomorphometrical analysis after explantation of the material with a focus on the analysis of parameters like bone formation, resorption rate, and contact between the implant and osseous tissue [18,43,51,52]. Barbieri et al. particularly focused on the surrounding tissue response (inflammation, soft tissue capsules and present cells) by implanting the material not only orthotopically into the bone but also in muscular pouches that were evaluated histologically [43]. To confirm and further detail the histologic results, Yu et al. and Bellucci et al. performed scanning electron microscopy (SEM), with a special focus on the cell–material interaction and surface alterations such as the formation of a HCA-layer (mentioned as bone-implant-interface or bone bonding mechanism) [18,51].

In the study of Bellucci et al., a post-mortem radiograph of the rabbit’s femur was conducted in order to evaluate the position of the implanted bone graft materials, as well as an X-ray energy disperse spectroscopy (EDS) assay with the aim to assess the dissolution kinetics of the implant by detection of silicon ions that are specific for the used 45S5-BG [18].

Barbieri et al. further performed calcein labeling to evaluate bone development and growth over the time, as calcein deposits in growing bone after intravenous injection and is detectable through fluorescence microscopy [43,80]. 

## 9. Biological Properties of Composite Materials

As mentioned above, high demands are placed on modern synthetic bone substitutes in order to reduce or even replace the usage of autologous bone graft material. Moore et al. therefore pointed out four crucial characteristics of such materials: osseointegration, osteoconduction, osteoinduction, and osteogenesis [81]. This basically means that the substitute should be able to bond to the surface of existing adjacent bone, to allow new bone to form on its surface, to induce differentiation of mesenchymal stem cells into cells of the osteoblastic lineage, and to let these osteoblasts form new bone on as well as within the material leading to step-by-step replacement of the substitute by newly formed host bone [81]. To complete this latter point, biodegradability should be added as a required feature. Degradation of bone substitutes occurs in form of dissolution as well as active cell-mediated resorption, mostly realized by osteoclasts [82,83]. Degradation is important not only in terms of replacement of foreign material by host tissue, but also the release of ions that are able to induce cell attachment, differentiation, and proliferation [16]. Both CaPs and BGs only partially fulfill these demands, especially when used separately [81].

The studies included in this meta-analysis either used in-vitro methods or in-vivo methods to assess the biological properties of the respective materials. Therefore, the following chapter is divided into an in-vitro and an in-vivo section and subdivided according to the methods used.

### 9.1. Cell Vitality (In-Vitro)

Looking at the included studies, cell vitality was the most investigated endpoint regarding biological properties, as 10 out of 14 studies analyzed it using different experimental approaches in-vitro. Cell vitality herein is meant as a collective term for viability, proliferation, number, and metabolic activity of cultured cells. Out of these 10 studies, seven directly compared CaP/BG composites to pure CaPs. Six of these studies revealed favorable results for CaP/BG composites [44,45,49,50,51]. They mostly used tetrazolium-based cell viability assays such as the MTT or the related 3-(4,5-dimethylthiazol-2-yl)-5-(3-carboxymethoxyphenyl)-2-(4-sulfophenyl)-2H-tetrazolium, inner salt (MTS) in order to determine the cell viability by measuring the metabolic activity of the cells. Under standardized conditions, one can also deduce the number of living cells in the sample. To give an example, Lopes et al. determined significantly higher amounts of cells on β-TCP scaffolds containing 7.5% 45S5-BG compared to those containing 5% or 0% using the MTT test, indicating that a higher amount of 45S5-BG supports the viability and proliferation of osteoblast-like cells [45]. This effect might be limited to a certain amount of 45S5-BG though. For instance, Bellucci et al. measured a continuous increase of cell quantity during cultivation time for their composite consisting of 20% 45S5-BG and 80% HA, whereas the composite containing 40% 45S5-BG and 60% HA showed a slight initial decrease of total cells during the first week of culture, followed by an increase until day 14 [48]. This indicates that too high amounts of 45S5-BG lead to a certain cytotoxicity, at least initially, which was also described in recent literature [41]. Interestingly, the other BG used in this study (BG_Ca) induced a constant increase of cell number for both 20% and 40% composites [48]. The major difference between 45S5-BG and BG_Ca is the alkali (Na_2_O) content (24.5% vs. 4.6%). The presence of sodium ions might be a possible explanation for these differences, especially since the dissolution behavior of the materials seems to correspond to pH measurements [48]. According to additional in-vitro results, there is growing evidence that alkali-reduced or -free BGs might be superior regarding osteogenic properties when compared to 45S5-BG [84].

This given explanation can be supported by the findings of Cholewa-Kowalska et al. as well as Hesaraki et al., due to the fact that the BGs used in the mentioned studies did not contain Na_2_O at all but showed significantly better results in terms of cell viability for CaP/BG compositions than for pure CaP [49,50]. While Cholewa-Kowalska et al. revealed the best results for 50% BG composites [49], the percentage of BG (10%, 25% and 40%) did not seem to have a major effect on cell proliferation in the study of Hesaraki et al. [50].

Conflictingly, Chen et al. used 45S5-BG at very high concentrations (93.3% of the composite), yet showed the best results for this composite compared to pure CaP and pure BG [44]. It should be mentioned though that only indirect cultivation approaches were used in this study (Figure 1). After immersion of the materials in cell culture medium, the enriched medium was diluted with DMEM, at a ratio of 33% in this particular attempt [44]. Furthermore, it is known that the influence on cellular metabolism differs from indirect to direct culture settings [58]. Therefore, the high initial content of BG in this study cannot be directly compared to those in the other studies. Regarding cell proliferation, a direct approach may reflect reality slightly better compared to an indirect one, since in a bone defect cells are supposed to be viable and able to proliferate in direct proximity to the bone substitute material. Furthermore, they ideally should even migrate into the material and eventually build new bone there. Therefore, concerning proliferation, the direct approach—in this matter used in most of the mentioned studies—seems rather convenient, since results can be transferred more directly to the in-vivo or even the anticipated clinical situation.

Cai et al. determined proliferation by directly counting cells in a blood cell counting chamber, also measuring higher cell numbers on CaP/BG composites compared to pure CaP [51].

In another study, Bellucci et al. compared HA/BG_Ca/Mix scaffolds not to pure HA, but to pure 45S5-BG, revealing comparable and in some cases even better results for the composites [19]. Haimi et al. also did not detect remarkable differences in cell viability and proliferation when comparing composite materials to pure BGs or CaPs [53]. In the study of Lu et al. CaP/BG was not compared to any other material regarding cell viability [46]. Therefore, a clear statement whether composites or pure materials are advantageous cannot be made. However, MTS tests showed a threefold increase in cell number between day one and seven of cell culture [46].

Only Bernhardt et al. presented several disadvantages of composites in terms of cell viability. At best, CaP/BG composites reached cell numbers comparable to those of pure β-TCP, falling behind with ongoing cultivation time [47].

Generally speaking, the rapid early dissolution of the BGs resulting in a major release of the ions correlating with excessive bioreactivity is a limitation for the use of BGs mainly in, but not limited to, in-vitro settings [41]. This rapid early release is often followed by an elevation of pH in the culture medium [48], inducing rather unfavorable conditions for cell cultivation [41]. However, a slightly alkaline surrounding favors the differentiation and activity of osteoblasts, whilst the function of osteoclasts, a cell type that is of certain relevance in the early phases of bone regeneration, is stimulated by slightly acidic surroundings [34,35,36]. There is limited evidence about the interaction of BGs with osteoclasts [83], but a dose-dependent enhancement of osteoclast differentiation was described in the presence of 45S5-BG [85]. By modulation of osteoclast activity, the degradation rate of mainly TCP-based composite materials might be modulated: Since TCP-based CaP-scaffolds tend to degrade (too) fast, adding BG in certain concentrations might slow down cellular degradation of the scaffold material [16,83]. However, further data analyzing the interactions of osteoclasts, osteoblasts, and CaP/BG composites is required to understand the underlying mechanisms [83].

In summary, according to most of the surveyed studies (92%), CaP/BG composites seem to have an advantageous or at least equal effect on in-vitro cell viability and proliferation compared to the respective materials alone.

### 9.2. Osteogenic Differentiation (In-Vitro)

Since CaP/BG composites are mainly designed for application in bone tissue engineering or bone regeneration, the influence of the material on osteogenic differentiation is an outcome parameter with great relevance. Therefore, in six studies either ALP activity assay, qPCR, RT-PCR, Alizarin Red S-staining, ALP staining or combinations of those were used [44,46,47,48,49,53]. In doing so, only Chen et al. realized an indirect approach since their focus lay on the addition of trace elements to the particular materials [44]. To give a counterexample, Bernhardt et al. focused more on the adhesion of cells, making direct approaches more suitable [47]. Four out of the six mentioned studies came to the conclusion that their particular composition of CaP and BG led to improved differentiation of MSCs or osteoblastic precursor cells towards osteoblasts [44,46,48,49]. All of these studies used assessment of ALP activity to determine osteogenic differentiation. In the studies of Chen et al., Lu et al., and Cholewa-Kowalska et al., compared to pure CaP increased ALP activity for CaP/BG composites within their individual periods of time were described [44,46,49]. Moreover, higher amounts of BG seemed to be advantageous compared to lower ones regarding ALP activity [49]. Bellucci et al. also reached higher levels of ALP activity for higher amounts of both BG_Ca and 45S5-BG within their composites, thereby excelling pure BGs [48].

Chen et al. and Lu et al. were also able to verify these results via qPCR by showing higher gene expression of osteogenic markers for composite groups than for pure CaP and BG respectively [44,46]. Examples for tested genes are ALP, Runx2 (Runt-related transcription factor 2), Col-I (collagen type I) and OPN (osteopontin), as well as other genes commonly associated with osteogenic differentiation [44,46]. Chen et al. even further confirmed their results on a “protein level”, describing higher amounts of Runx2, Col-I and OC (osteocalcin) for the composite compared to pure CaP as well as pure BG [44].

At first sight, the study of Bernhardt et al. seems to describe contradictory findings concerning osteogenic differentiation: ALP activity in pure CaP was at least as high as or, depending on the point in time, even significantly higher than in CaP/BG [47]. Yet, a closer look reveals that the promising results of the other studies were often reached at BG percentages as high as 40% or even 50% [48,49], whereas Bernhardt et al. used a composition with only 4% BG [47]. For example, Cholewa-Kowalska et al. obtained similar values for pure CaP, pure BG and 10% BG composites, but significantly higher values for composites containing 50% BG [49]. This might reflect the need for a certain minimum content of BG that is necessary to alter the osteogenic properties of composite materials. At the same time, Bellucci and coworkers showed that composites might also outreach pure BGs [48], again indicating a certain suitable maximum of BG content.

However, Haimi et al. revealed no significant differences between pure BG and CaP-coated BG [53]. Interestingly though, osteopontin levels measured in the culture medium was significantly higher for samples with thin CaP-coating than for those with a thick coating [53]. This indicates that the amount of time a respective bone substitute material is preliminarily exposed to physiological conditions correlates with its osteoinduction and thus its biological properties. However, this may only occur due to the fact that under a thick layer of CaP the beneficial properties of the BG do not sufficiently affect the behavior of cultivated cells.

### 9.3. Microscopy (In-Vitro)

Another methodology often used in the included studies was conventional and/or scanning electron microscopy (SEM). These methods are often confined to rather qualitative statements, since standardization and technical feasibility can limit substantiated quantification. Nevertheless, microscopy often provides important impressions about basic mechanisms and might be used to qualitatively confirm statements acquired by other methods regarding plausibility. Three out of six studies that used microscopy in-vitro described promising results when using CaP/BG composites [46,48,51], whereas the other half revealed no major difference between the compositions and pure CaP [47] or between composites with different amounts of BG or CaP respectively [19,53].

Lu et al., Cai et al. and Bellucci et al. all describe good cell adhesion and distribution on CaP/BG scaffolds by virtue of their SEM findings [46,48,51]. For example, Cai et al. revealed an almost continuous layer of osteoblasts on the β-TCP/BG mixture, whereas this observation was not made on pure β-TCP [51]. This indicates that phase composition is a crucial feature of bone substitute materials when it comes to cell adhesion, proliferation, and—as the desired consequence—new bone formation. It should be mentioned though, that different melting behavior during the fabrication of the scaffolds might lead to different microporosity and therefore different surface properties on the struts on a micron range [51]. These surface properties however are important characteristics of bone substitutes regarding cell adhesion, as osteoblasts and MSCs respectively prefer a rather rough surface [16,48].

In contrast, according to Bernhardt et al. β-TCP and β-TCP/BG scaffolds showed no remarkable difference regarding cell adhesion [47]. Notably, they used 4% BG in their composition, whereas Cai et al. and Bellucci et al. used 20% and up to 40% respectively [48,51]. This again leads to the implication that higher contents of BG may favor attachment, distribution, and—as already concluded above—viability, proliferation, and differentiation of cells. In another study, Bellucci et al. analyzed composites consisting of 30% as well as 80% BG with the appropriate amounts of HA, showing no major histomorphometric difference between these two concentrations [19]. Comparable results were revealed by Haimi et al., who showed no qualitative differences in cell morphology and proliferation on either pure BG and different CaP-coated BGs according to their SEM investigations [53]. Nevertheless, they described a sort of active contact between cells and BG, appearing as “cellular bridges” between BG particles [53].

Hence, although BG seems to support cell attachment and distribution, declaration of a suitable amount of BG for optimal in-vitro cell behavior seems to require further research.

### 9.4. Microscopy (In-Vivo)

As mentioned before, four out of 14 studies used in-vivo models. One major benefit, especially for bone defect models, is that these models provide a physiological surrounding for the bone substitute, so the model mimics the actual clinical situation of a bone defect [15]. Ossoeintegration, bone formation within the implant, and resorption kinetics can all be qualified and quantified by microscopy approaches. Although all four mentioned studies were conducting microscopy, they show rather heterogeneous results. Histomorphometry remains one of the most relevant methods in in-vivo studies for tissue discrimination and can even be used to quantify bone volume under certain conditions [7,86]. By specialized staining, such as antibody-based immunohistology or histochemical approaches, distinct properties that are affiliated with bone substitute materials can be assessed, for example the angiogenic potential [87]. Unfortunately, none of these approaches were used in either of the analyzed in-vivo studies.

Bellucci et al. described that according to histomorphometry, increased amounts of BG lead to increased osteoconduction, thus new bone formation and the ability of newly formed bone to attach to the surface of the implant [18]. In contrast to these findings, Barbieri et al. could hardly detect any newly formed bone on CaP/BG scaffolds, whereas bone formation on CaP scaffolds was better [43]. Notably though, the scaffolds investigated consisted not only of CaP, but also of an AOC [43]. Since there was no control group represented by pure CaP, it remains unclear whether bone formation was induced by CaP or AOC. For CaP/BG compositions, new bone formation could be detected only on neighboring host bone [43].

Kucukkolbasi et al. as well as Yu and coworkers did not mention distinct differences between pure CaP and CaP/BG composites in terms of new bone formation or connective or marrow tissue [51,52]. Interestingly, the former of the two reported best results for a combination of HA, BG, and demineralized freeze-dried bone [52]. Considering the enormous plurality of possible additional composite materials, their further discussion would go beyond the scope of this review.

In summary, in-vivo investigations of bone substitute materials suggest that BG may support bone formation and healing under certain circumstances [18] as well as in presence of host bone [43]. Interestingly, the addition of further materials might also enhance the osteogenic properties of such materials [43,52].

## 10. Conclusions

BGs are known to stimulate osteogenic differentiation of precursor cell populations and are able to bond to surrounding tissues helping to integrate BG-based bone substitutes into bone, which is mediated by their surface reactivity. However, BGs suffer from poor mechanical properties when used as 3D bone substitutes. Furthermore, the local pH-changes in the BG environment can be harmful to cells. CaPs, the most commonly used bone substitute materials, show good osteoconductive properties, but the material itself induces only limited stimulation of osteogenic differentiation and surface reactivity is comparably low. Not only the material properties but also the biological and osteogenic properties might be improved and the individual limitations of the materials might be overcome by combining CaPs and BGs, creating CaP/BG composite materials. In this review paper, the available data analyzing the biological properties of CaP/BG composite materials and the impact of BG addition to the properties of CaPs was summarized and evaluated. Since the collective of studies analyzing the impact of BG on the biological properties of the materials was very heterogenous, direct comparison of the studies´ results was not possible. However, when analyzing the studies individually, tendencies were detectable, especially in studies executing direct comparison of BG-supplemented and BG-free CaPs. In conclusion, BG-addition has positive effects on cell adhesion, viability, and proliferation compared to pure CaP materials. In-vivo, the presence of BG supports integration of the materials into bone as well as enhancing bone formation under certain circumstances. Future studies should analyze the effects of the composite materials on in-vivo resorption kinetics, since inappropriate degradation kinetics are a limitation of pure CaPs that can be tailored by addition of BGs. In order to do that, not only chemical but also cellular degradation should be assessed, meaning the interaction of BGs, CaPs, and CaP/BG composites with resorbing cells such as osteoclasts, which is not yet well understood. Direct comparison of the studies’ outcomes was difficult due to the heterogeneous study designs. Therefore, upcoming studies should further commit to use comparable experimental setups in order to make inter-study interpretation of results feasible and to further support understanding of the biological properties of different CaP/BG composites.

## Figures and Tables

**Table 1:** Studies included in the review.

First Author	Ref.	Year	Composite		Percentage		Setup	Methods/Endpoints	Biological Outcome
			1st Phase	2nd Phase	1st Phase	2nd Phase			
Barbieri	[43]	2017	HA/β-TCP 4/96	AOC	51.7	48.3	in-vivo	Histomorphometry	Compared to CaP/AOC rarely any formation of new bone on CaP/BG composite.
			β-TCP	45S5 (45 wt% SiO_2_, 24.5 wt% CaO, 24.5 wt% Na_2_O, 6 wt% P_2_O_5_)-Collagen mix	61.8	38.2			
Bellucci	[18]	2017	HA	BG_Ca/Mix (47.3% SiO_2_, 45.6% CaO, 2.3% K_2_O, 2.3% Na_2_O, 2.6% P_2_O_5_)	70/20/0	30/80/100	in-vivo	X-Ray, histomorphometry	Increased osteoconductivity of pure BG scaffolds compared to composites, with best results for BG_Ca/Mix. Improved osteoconductivity of CaP/BG composites with increasing BG content.
			45S5	/	100	0			
Chen	[44]	2017	Si-Sr-Zn-Mg-codoped CaP	45S5	100/6.7/0	0/93.3/100	in-vitro	Cell proliferation, osteogenic differentiation, protein expression	Improved proliferation and differentiation of mesenchymal stem cells in composite materials.
Lopes	[45]	2016	β-TCP	45S5	100/95/92.5	0/5/7.5	in-vitro	Cell viability, SEM	Increased cell viability and advanced attachment in 7.5%-composites.
Bellucci	[19]	2015	HA	BG_Ca/Mix	20/70	80/30	in-vitro	Cell viability, cell proliferation, cytotoxicity	Cell viability in composites equivalent to 45S5-BG.
			45S5	/	100	0			
Lu	[46]	2015	HA/β-TCP (40/60)	58S (60% SiO_2_, 36% CaO, 4% P_2_O_5_)	n/s	n/s	in-vitro	Cell viability, osteogenic differentiation	Synergistic effect of BG and CaP on osteogenic differentiation, further enhanced by BMP-2.
			HA/β-TCP (40/60)	/	100	0			
Bernhardt	[47]	2013	β-TCP	Na-Mg-Si-BG system	96	4	in-vitro	Cell adhesion, osteogenic differentiation, cell viability	Cell viability and number increased or equal for pure β-TCP vs. β-TCP/BG composites. No differences in osteogenic performance (ALP activity) for pure β-TCP vs. β-TCP/BG after 28 days of incubation. Unless pre-incubated, HA/β-TCP/BG composites and HA/BG composites did reduce cell number compared to pure β-TCP.
			HA/β-TCP (60/40)	SiO_2_ matrix	n/s	n/s			
			HA	SiO_2_ matrix	76	24			
			β-TCP	/	100	0			
Bellucci	[48]	2013	HA	BG_Ca (47.3% SiO_2_, 45.6% CaO, 4.6% Na_2_O, 2.6% P_2_O_5_)	80/60	20/40	in-vitro	Cell adhesion, cell proliferation, cell viability, osteogenic differentiation	Increased osteogenic differentiation for BG_Ca composites (ALP activity) vs. 45S5-BG composites. Increased osteogenic differentiation with increasing BG content in both composites. No major differences in cell proliferation between the different composites.
				45S5					
Cholewa-Kowalska	[49]	2009	HA	S2 (80% SiO_2_, 16% CaO, 4% P_2_O_5_)	100/90/50/0	0/10/50/100	in-vitro	Cell viability, osteogenic differentiation	Osteogenic differentiation (ALP activity) significantly improved for the 50:50 S2 composite.
				A2 (40% SiO_2_, 54% CaO, 6% P_2_O_5_)					
Hesaraki	[50]	2009	β-TCP	64% SiO_2_, 26% CaO, 5% P_2_O_5_, 5% MgO	90/75/60	10/25/40	in-vitro	Cell viability	Improved cell viability in composite materials, regardless of BG content.
Yu	[51]	2009	β-TCP	62.04 wt% P_2_O_5_, 14.68 wt% CaO, 13 wt% MgO, 10.28 wt% Na_2_O	100/80	0/20	in-vivo	SEM, histomorphometry	No differences in in-vivo biocompatibility between pure β-TCP and composite material.
Kucukkol-basi	[52]	2009	HA	45S5	0/50/100	100/50/0	in-vivo	Histomorphometry	No major differences between the composite and both pure HA and 45S5-BG.
Cai	[51]	2009	β-TCP	45 wt% P_2_O_5_, 22 wt% CaO, 25 wt% Na_2_O, 8 wt% MgO	100/80	0/20	in-vitro	SEM, cell count	BG addition increased cell number as well as attachment and distribution of cells.
Haimi	[53]	2009	CaP (n/s)	Na_2_O, K_2_O, MgO, CaO, B_2_O_3_, TiO_2_, Ca_2_PO_5_, SiO_2_	n/s	n/s	in-vitro	Cell adhesion, cell viability, cell proliferation, osteogenic differentiation	No remarkable differences between pure BG and CaP-coated BG.

β-TCP: β-tricalcium phosphate, AOC: Alkylene oxide copolymer, n/s: not specified, % = mol% unless stated otherwise, wt% = percentage by weight. Methods = biological methods, SEM: scanning electron microscopy, ALP: alkaline phosphatase. When glass compositions are linked to names (e.g., 45S5) the exact composition is shown when mentioned first.

**Table 2:** In-vitro evaluation, study designs.

First Author	Ref.	Cell Type	Species	Cell Number	Medium	Culture Time	Culture Setting
Chen	[44]	rMSC/rOMSC	rat	100,000/cm^2^	MM + Osteogenic factors (β-GP + AA)	21 d	indirect
Lopes	[45]	MG 63	human	8000/well (96-well-plate)	MM	3 d	direct
Bellucci	[19]	BALB/3T3 + MLO-Y4	mouse	n/s	MM	24 h–48 h	direct and indirect
Lu	[46]	ASC	human	50,000/0.6 cm^2^/10,000/0.6 cm^2^	MM	14 d	direct
Bernhardt	[47]	SaOS-2	human	160,000/0.1 2 cm^3^	MM + Osteogenic factors (β-GP + AA)	28 d	direct
Bellucci	[48]	MC3T3-E1	mouse	50,000/g	GM + ODM (including β-GP + AA)	21 d	direct
Cholewa-Kowalska	[49]	hBMSC	human	10,000/cm^2^	MM + ODM (including DM + AA)	7 d	direct
Hesaraki	[50]	G-292	human	30,000/mL	MM	7 d	direct
Cai	[51]	MC3T3-E1	mouse	1,000,000/0.375 cm^2^	MM	14 d	direct
Haimi	[53]	ASC	human	500,000/0.98 cm^2^	MM	14 d	direct

n/s: not specified, rMSC: rat bone marrow-derived mesenchymal stem cells, rOMSC: osteoporotic rat bone marrow-derived mesenchymal stem cells, MG 63: osteoblast-like cells, BALB/3T3: mouse embryonic fibroblast cell line, MLO-Y4: murine long bone osteocyte-like immortalized cell line, ASC: adipose tissue-derived mesenchymal stem cells, SaOS-2: osteoblast-like cells, hBMSC: human bone marrow stromal cells, G-292: human osteosarcoma cells, MC3T3-E1: mouse osteoblast precursor cell line, MM: maintenance medium, GM: growth medium, ODM: osteogenic differentiation medium, β-GP: β-glycerophosphate, AA: ascorbic acid, DM: dexamethasone. The cell number per sample refers either to the surface of the scaffold/the well (cells/cm^2^) or to the scaffold without further specification (arbitrary unit) or the volume of the medium (cells/mL). Culture time in days (d) or hours (h). If multiple cell numbers are indicated, different cell numbers were used for different assays within one study.

**Table 3:** In-vivo evaluation designs.

First Author	Ref.	Species	Location and Defect Size	Implantation Time	Methods	Parameter
Bellucci	[18]	New Zealand Rabbit	Femur (3.5 mm diameter, 7 mm depth)	2 m	Post-mortem femur X-ray	Graft position
					Histomorpho-metry	Implant-bone-interface: bone formation, bone healing/contact, cracks of the graft
					SEM	Bioactive bone bonding mechanism (confirmation of histology) HA-Layer
					EDS + microradiography	Dissolution kinetics
Barbieri	[43]	Mongrel dog	Orthotopic (spine) and ectopic (i.m.) (defect size n/s)	3 m	Histomorpho-metry	Bone formation, bone-surface-contact, in-vivo resorption rate tissue response (i.m.)
					Fluorescence microscopy	Bone development (calcein labeling)
Yu	[51]	New Zealand Rabbit	Femur (2 mm diameter, depth n/s)	1/2/3/6 m	Histomorpho-metry	Bone formation, bone-implant-interface, resorption rates
					SEM	Bone formation, bone-implant-interface, resorption rates
Kucukkolbasi	[52]	New Zealand Rabbit	Tibia (3 mm diameter, depth n/s)	1/3/6 m	Histomorpho-metry	Bone formation, resorption rates, tissue reactions

SEM: scanning electron microscopy, EDS: energy dispersion spectroscopy, HA: hydroxyapatite, i.m.: intramuscular. Implantation time in months (m).

## Abbreviations

2D	two-dimensional
3D	three-dimensional
AA	ascorbic acid
ALP	alkaline phosphatase
AOC	alkylene oxide copolymer
ASC	adipose tissue-derived mesenchymal stem cells
BALB/3T3	mouse embryonic fibroblast cell line
BG	bioactive glass
CaP	calcium phosphate
Col-I	collagen type I
DM	dexamethasone
DMEM	Dulbecco’s modified eagle medium
EDS	energy dispersion spectroscopy
ELISA	Enzyme-linked Immunosorbent Assay
G-292	human osteosarcoma cells
GM	growth medium
HA	hydroxyapatite
hBMSC	human bone marrow stromal cells
HCA	hydroxyapatite-like
i.m.	intramuscular
LDH	lactate dehydrogenase
MC3T3-E1	mouse osteoblast precursor cell line
MG 63	osteoblast-like cells
MLO-Y4	murine long bone osteocyte-like immortalized cell line
MM	maintenance medium
MSC	mesenchymal stem cells
MTT	3-(4,5-dimethylthiazol-2-yl)-2,5-diphenyltetrazolium bromide
MTS	3-(4,5-dimethylthiazol-2-yl)-5-(3-carboxymethoxyphenyl)-2-(4-sulfophenyl)-2H-tetrazolium, inner salt
n/s	not specified
Na_2_O	alkali
NRU	Neutral Red Uptake Test
OC	osteocalcin
ODM	osteogenic differentiation medium
OP	osteopontin
qPCR	quantitative real-time PCR analysis
rMSC	rat bone marrow-derived mesenchymal stem cells
rOMSC	osteoporotic rat bone marrow-derived mesenchymal stem cells
RT-PCR	Reverse transcriptase PCR
Runx2	Runt-related transcription factor 2
SaOS-2	osteoblast-like cells
SEM	scanning electron microscopy
TCP	tricalcium phosphate
XTT	2,3-bis(2-methoxy-4-nitro-5-sulfophenyl)-5-[(phenylamino)carbonyl]-2H-tetrazolium hydroxide
β-GP	β-glycerophosphate
